# Prevalence and treatment response of neuropsychiatric disorders in mast cell activation syndrome

**DOI:** 10.1016/j.bbih.2025.101048

**Published:** 2025-06-30

**Authors:** Leonard B. Weinstock, Lawrence B. Afrin, Angela M. Reiersen, Jill Brook, Svetlana Blitshteyn, Gillian Ehrlich, Jill R. Schofield, Laurence Kinsella, David Kaufman, Tania Dempsey, Gerhard J. Molderings

**Affiliations:** a Gastroenterology Department, Gastrointestinal Alliance, 11525 Olde Cabin Road, St. Louis, MO, 63141, USA; b AIM Center for Personalized Medicine, Senior Consultant in Hematology/Oncology, Department of Mast Cell Studies, 3010 Westchester Avenue, Suite 404, Armonk, NY, 10577, USA; c Washington University in St. Louis School of Medicine, Department of Psychiatry Box 8134, 660 S Euclid Ave, St. Louis MO, 63110, USA; d PatientsCount.org, 13285 Roundhill Drive, Truckee, CA, 96161, USA; e Clinical Associate Professor of Neurology, University at Buffalo Jacobs School of Medicine and Biomedical Sciences, Buffalo, NY 14203, USA; f Neuroveda Health, 1700 Westlake Ave N, Suite 100, Seattle, WA, 98109, USA; g University of Colorado Anschutz School of Medicine, 13001 E 17th Pl, Aurora, CO, 80045, USA; h SSM Health St Clare Neuroscience Institute, Adjunct Professor in Neurology, Saint Louis University, Fenton, MO, 63390, USA; i Center for Complex Diseases, 2206 Queen Anne Ave. N, #303, Seattle, WA, 98109, USA; j AIM Center for Personalized Medicine, Department of Integrative Medicine, 3010 Westchester Avenue, Suite 404, Armonk, NY, 10577, USA; k University Hospital of Bonn, Institute of Human Genetics, Venusberg-Campus 1, D-53127 Bonn, Germany

**Keywords:** Mast cell activation syndrome, MCAS, Neurologic, Psychiatric

## Abstract

**Background:**

Neuropsychiatric disorders have been observed in mast cell activation syndrome (MCAS). MCAS is a common, yet rarely diagnosed, inflammatory, and immunologic disease characterized by mast cell dysregulation.

**Methods:**

Questionnaires from 553 MCAS and 558 control subjects determined the prevalence and odds ratio of neurologic disorders (fatigue, cognitive dysfunction, fainting/near fainting, migraine-like headaches, muscle pain/tenderness/weakness, pain/numbness/tingling in extremities, restless legs syndrome, seizure-like activity, insomnia, sleep attacks, tinnitus, acoustic startle, Tourette's syndrome, resting tremor, and light/sun/pain/odors/scents/noise hypersensitivity) and psychiatric disorders (anxiety, agoraphobia, panic attacks, depression, bipolar depression, mania/hypomania, psychosis/schizophrenia, hallucinations, obsessive compulsive disorder, attention-deficit/hyperactivity disorder, anger management problems, post-traumatic stress disorder, suicidal thoughts, and eating disorders).

**Results:**

Among 19 neurologic disorders, female MCAS patients reported higher rates in all but 1 disorder and male MCAS patients reported higher rates in all but 2 disorders. Among 14 psychiatric disorders, female MCAS patients reported higher rates in all and male MCAS patients reported higher rates in 8 disorders. Many of the disorders with increased prevalences were statistically greater compared to corresponding controls.

In self-reported ratings for effects on health status (0 = no benefit, 10 = maximum benefit), mean (SD) response was 6.3 (2.5) for antihistamines, 5.6 (3.2) for low-dose naltrexone, and 5.6 (3.1) for benzodiazepines.

**Conclusion:**

MCAS subjects have significantly elevated odds ratios for many neuropsychiatric disorders and may see improvement of symptoms using MCAS-targeted therapies, suggesting that mast cell dysregulation affects the brain and peripheral nervous systems and contributes to neuropsychiatric symptoms. Certain mast cell mediators, specific genetic predisposition, and life experiences could determine which disorder is apt to develop or worsen.

## Highlights

• MCAS is a common inflammatory disease characterized by mast cell dysregulation.
• 19 neurologic and 14 psychologic disorders were significantly increased in MCAS subjects.
• Chemical mediators, genetic predisposition, and life experiences could determine which disorders occur or worsen.

## 1 Introduction

### 1.1 Overview of treatment of neuropsychiatric disorders

The clinical and societal impacts of neuropsychiatric (NP) disorders are profound, affecting countless individuals and families (Hill et al., 2021; Barr et al., 2022). Therefore, it is crucial to pursue treatments that specifically target the underlying pathophysiology. The impact of NP disorders is especially substantial for migraines, cognitive dysfunction, autism, restless legs syndrome (RLS), and depression (Burch et al., 2018; Hale et al., 2020; Theoharides et al., 2012a, Theoharides et al., 2012b, Theoharides et al., 2012c-a; Allen et al., 2005; Barr et al., 2022). These disorders and others have been associated with neuroimmune dysfunction and can be difficult to treat (Liberman et al., 2018).

### 1.2 Multisystemic presentations of mast cell activation syndrome

Mast cell activation syndrome (MCAS) remains unrecognized as the primary cause for many complex patients with a multisystemic disorder (Afrin et al., 2016-a; Afrin et al., 2016-b; Afrin et al., 2020a, Afrin et al., 2020b). Symptoms of MCAS typically begin during childhood, sometimes in infancy (Afrin et al., 2020a, Afrin et al., 2020b). Patients may experience a variety of non-specific gastrointestinal and dermatologic symptoms, headaches, and frequent episodes of inflammation, which may be misinterpreted as infections. Other issues can include developmental anomalies, problems with the integrity and healing of connective tissues, and atopic disorders. MCAS-associated NP manifestations can be caused by dysfunction of the abnormal MCs in the central and/or peripheral nervous system or indirectly by MC mediators in other tissues which lead to inflammatory and other effects in nervous systems (Afrin et al., 2015). For instance, cytokines such as IL-12, a key mediator of neuroinflammation, may contribute to NP symptoms by altering neurotransmitter systems and activating microglia (Chauhan et al., 2025). While NP manifestations have been reported in systemic mastocytosis (SM), a rare malignancy, MCAS itself has only recently gained attention, making it an underappreciated and often treatable differential diagnosis to explain a wide range of NP disorders (Sagües-Sesé et al., 2023; Afrin et al., 2015). Many NP disorders may stem from this more prevalent MC disease. Inflammatory disorders are often more prominent than allergic phenomena. Although allergic, dystrophic, and other phenomena are commonly seen in MCAS, chronic multisystem inflammation is the universal constant, or sine qua non, of MCAS (Afrin et al., 2016-a). Concordant with the MC's evolutionarily defined role as the immune system's front-line sentinel against insults to the body, a very wide range of stimuli for triggering MC activation has been identified, including a vast array of physical substances and forces (Weinstock et al., 2024). Mast cell activation and NP symptoms are known to be worsened by stress in humans and in lab animals (Theoharides, 2000; Sanacora et al., 2022). Thus, in MCAS there is not only constitutive chronic aberrant mediator release by the dysfunctional MCs but also inappropriate reactive mediator release upon exposure to various triggers.

The diagnosis of MCAS is often delayed for decades. Reasons for this include: 1) the relative absence of MCAS education in medical school and residency curricula, 2) the common misconception that an increased serum tryptase level is needed to establish the diagnosis, and 3) the marked heterogeneity of the disease's clinical presentation (consequential to its marked heterogeneity at the genetic mutation level and the mediator expression level).

One should consider a differential diagnosis to include systemic mastocytosis and subclassifications, monoclonal MCAS, and secondary MCAS. As is the case with most challenges in differential diagnosis, it is the totality of the patient's presentation (the symptoms and problems experienced throughout the patient's life, and the full extent of findings on comprehensive physical examination and thorough laboratory testing including properly technically executed testing looking for elevated levels in blood or urine of mediators relatively specific to the mast cell) which fairly reliably reveals the patients in whom MCAS is most likely underlying/unifying diagnosis accounting for the largest extent of the problems and findings in the patient to date.

### 1.3 Etiology and prevalence of mast cell activation syndrome

Mast cell activation syndrome (MCAS) is characterized by unregulated immune responses often linked to genetic mutations in mast cell (MC) regulatory genes (Molderings et al., 2007, Afrin et al., 2016a; Afrin et al., 2017; Afrin et al., 2020a, Afrin et al., 2020b). Epigenetic changes leading to somatic variants in MC regulatory genes are usually at the root of this lifelong illness (Molderings, 2007, 2022). Such variants and consequential MC dysfunction usually begin early in an MCAS patient's life. As most MCAS patients progress through life, the acquisition of other variants (typically soon after major stressors) often leads the disease to escalate its baseline level of MC dysfunction, potentially affecting any or every system in the body (Afrin et al., 2020a, Afrin et al., 2020b).

MCAS is diagnosed far more commonly in females than males (∼4–5:1), which may be due to biological determinants (e.g., MC-surface estrogen receptors) (Zierau et al., 2012). The prevalence of MCAS in the Northern Hemisphere is estimated at 4 %–17 % (Zaghmout et al., 2024; Molderings et al., 2013).

The biology and anatomical distribution of MCs in proximity to peripheral and central neurons may account for a wide range of extensive NP symptoms seen in these patients (Theoharides et al., 2024). Hundreds of mediators and cell-surface receptors are known to be expressed by mast cells (MCs) (Molderings, Afrin, 2023). In the largest series of prospective MCAS patients (N = 413), the prevalence of various NP disorders and symptoms included fatigue (83 %), fibromyalgia-type pain (75 %), pre-syncope/syncope (71 %), headaches (63 %), cognitive dysfunction (49 %), insomnia (35 %), vision abnormalities (30 %), anxiety and/or panic attacks (16 %), depression (13 %) and involuntary movements (13 %) (Afrin et al., 2017). In 2010, an article listed headache, syncope, and psychiatric conditions that were associated with mast cell activation (Akin et al., 2010).

### 1.4 The brain, mast cells, mediators, and inflammation

MCAS-associated NP manifestations can be caused by dysfunction of abnormal MCs in the central and/or peripheral nervous system or indirectly by MC mediators in other tissues, leading to inflammatory and other effects in the peripheral and central nervous systems (CNS) (Theoharides et al., 2012a, Theoharides et al., 2012b, Theoharides et al., 2012c-b; Theoharides et al., 2016, 2017, 2019, 2024). Migraine is a common comorbidity noted in patients with MCAS (Blitshteyn, 2023). MCs are present in the meninges and are implicated in the pathophysiology of migraine via neuropeptide release, vasodilation, plasma, and protein extravasation that can lead to MC degranulation (Hendriksen et al., 2017). Degranulation of meningeal MCs may sensitize trigeminal vascular afferent processing (Blitshteyn, 2023). This MC-mediated pathway is thought to be one of the mechanisms underlying migraine pain pathophysiology (Aich et al., 2015). Additionally, circulating autoantibodies could affect the brain and autonomic nervous system due to an MC-induced hyperpermeable blood-brain barrier (BBB) and/or an abnormal functioning blood-cerebrospinal fluid barrier (Shelestak et al., 2020; Gelb, 2018). The role of MC activation in a variety of NP disorders has been studied in humans and in animal models (Theoharides et al., 2016; Moura et al., 2011; Georgin-Lavialle et al., 2016; Kempuraj et al., 2017; Nicoloro-SantaBarbara et al., 2021; Nicoloro-SantaBarbara et al., 2022; Jendoubi et al., 2021; Bidri et al., 1999; Ikarashi, Yuzurihara, 2002). Magnetic resonance imaging (MRI) has demonstrated morphological and functional abnormalities in the brains of SM patients with NP complaints, (Boddaert et al., 2017). MCAS patients with NP complaints also have similar neuro-radiographic findings (abnormal punctuated white matter abnormalities) (Haenisch, Molderings, 2018).

### 1.5 Inflammation, allergy, and psychiatric disorders

The field of immuno-psychiatry has been suggested as an agenda for clinicians to develop innovative research (Leboyer et al., 2016). Immune and/or inflammatory disorders have been suspected to play direct roles in the pathophysiology of the following disorders: pediatric autoimmune neuropsychiatric disorders associated with depression, streptococcal infection, pediatric acute-onset neuropsychiatric syndrome, bipolar depression, obsessive-compulsive disorder (OCD), and tic disorders (Leonard et al., 2001; Hamdani et al., 2012; Chang et al., 2015; Gerentes et al., 2019; Hsu et al., 2021; Marazziti et al., 2023). In an exceptionally large pediatric study of tic disorders (N = 4508), ADHD (N = 83,569), and/or OCD (N = 1555), there was a higher prevalence of atopy in tic disorders (51.6 %), attention-deficit/hyperactivity disorder (ADHD) (50.7 %), and OCD (47.7 %) compared to 75,000 controls (38.6 %) (Hakimi et al., 2022). The prevalence of allergic conjunctivitis, allergic rhinitis, asthma, and atopic dermatitis was higher in children with tics, ADHD, or OCD as compared to controls.

### 1.6 Mast cell therapy and improvement in neuropsychiatric manifestations

There is emerging evidence that MC therapy may be effective for NP disorders in patients with MCAD. Antihistamines have been recognized to be helpful in some NP disorders, including migraine and neuropathic pain (Worm et al., 2019; Khalilzadeh et al., 2018). Omalizumab was partially effective for several NP symptoms in SM and MCAS (Lemal et al., 2019). In MCAS, NP symptoms improved in a patient with severe MCAS and postural orthostatic tachycardia syndrome (POTS) using low-dose naltrexone (LDN), intravenous immune globulin, and antibiotic therapy for small intestinal bacterial overgrowth (Weinstock et al., 2018). A case series of 116 MCAS patients treated with LDN demonstrated improvement in a wide array of NP disorders (Weinstock, 2020-a). In another case series, combinations of antihistamines, LDN, and other MC medications were successful in treating standard-treatment refractory NP manifestations (Weinstock et al., 2023). An animal study showed overall improvement in hippocampal neuroinflammation, apoptosis and synapse dysfunction using zileuton (a leukotriene synthesis inhibitor occasionally used in MCAD) (Liu et al., 2020). Several patients who were first evaluated by the authors noted that chronic benzodiazepines appear to have had been prescribed by other doctors for anxiety and insomnia. These two conditions were the most common NP disorders seen in MCAS patients (Afrin et al., 2017). In the authors’ experience, benzodiazepines, typically at low, non-addicting doses and principally via their engagement with inhibitory benzodiazepine binding targets known to be present in MCs, not uncommonly help diverse MCAS symptoms including a variety of NP and systemic symptoms, not only when taken reactively during a trigger-induced symptomatic flaring of the disease but also when taken preventively at a low dose. An intravenous protocol that includes lorazepam appeared to help MCAS-induced gastrointestinal attacks (Weinstock et al., 2024). In vitro studies showed that benzodiazepines reduce MC activity (Birdri et al., 1999; Hoffmann et al., 2013; Yousefi et al., 2013; Haenisch et al., 2013). Luteolin, a flavonoid with anti-inflammatory efficacy, is used to treat MCAS as a MC stabilizer but has also been shown to improve autism spectrum disease, fatigue, and cognitive dysfunction (Theoharides et al., 2012a, Theoharides et al., 2012b, Theoharides et al., 2012c-c; Theoharides et al., 2012a, Theoharides et al., 2012b, Theoharides et al., 2012c; Tsilioni I, Theoharides, 2024; Gelabert-Rebato et al., 2019). Vitamin D is another MC stabilizer and is also protective against NP disorders (Wassif et al., 2023). Certain probiotics and herbal medications can also suppress MC-induced inflammation (Dev et al., 2008; Li et al., 2019).

### 1.7 Purpose of this study

The study sought to determine the prevalence of a variety of NP manifestations in MCAS patients in comparison to healthy controls. Chronic fatigue, cognitive dysfunction, faint/near faint, migraine-like headaches, muscle pain/tenderness, muscle weakness in the arms/legs, nerve pain/numbness/tingling, RLS, seizure-like activity, insomnia, sleep attacks, tinnitus, acoustic startle, Tourette's syndrome, resting tremors, and hypersensitivity to (a) light/sun, (b) pain, (c) odors/scents, or (d) noise were studied. Participants with MCAS were evaluated retrospectively for the historical efficacy and side effects of MC therapy by antihistamines, LDN, and benzodiazepines.

## 2 Methods

### 2.1 Study approval

The study was reviewed by the Sterling Investigational Review Board in Atlanta, Georgia. The board determined that the study (ID #12217, Protocol #12212) was exempt from full review pursuant to the terms of the U.S. Department of Health and Human Service's Policy for Protection of Human Research Subjects at 45 C.F.R. §46.104(d). The board determined that the exemption Category 2 applied. All participants signed an electronic informed consent form that allowed anonymous collection and reporting of clinical data.

### 2.2 Inclusion and exclusion criteria

MCAS subjects were included if they were previously diagnosed with MCAS by one of the investigators using either the consensus-1 and/or consensus-2 proposals for diagnostic criteria for MCAS (Afrin et al., 2020b, Afrin et al., 2020a). Consensus-1 and -2 criteria include 2 or more systems with MC symptoms, although anaphylaxis is usually present in consensus-1. Laboratory support for a diagnosis of MCAS per consensus-1 requires an increase of tryptase during a MC attack, whereas any increase of tryptase (not attributable to hereditary alpha-tryptasemia) or other mediators relatively specific to the mast cell supports a diagnosis of MCAS per consensus-2. The third criteria are similar in both consensus groups (required in consensus-1, though optional in consensus-2 in recognition of the disease's heterogeneity) – namely, that MC directed therapy improves the symptoms.

The study was a multicenter study with participants recruited from practices based in Missouri, New York, Washington, and Colorado. These clinicians had expertise in MCAS with specialties in gastroenterology (LW), hematology/oncology (LA), neurology (SB, LK), and internal medicine with a focus on complex diseases (GE, JS, TD, DK).

Control subjects were included if they had never been diagnosed with MCAS. Owing to the high prevalence of MCAS in the general population and significant genetic predisposition, it is possible that some of the controls had undiagnosed MCAS. All participants were asked if there was a personal or family history of anaphylaxis, atopic disorders (“severe allergic symptoms and/or asthma requiring medicines on a weekly basis year-round”), chronic fatigue syndrome (CFS), chronic pelvic pain syndromes (CPPS including interstitial cystitis, chronic prostatitis, and vulvodynia), chronic vomiting syndrome (CVS), Ehlers-Danlos syndrome (EDS), fibromyalgia syndrome (FMS), irritable bowel syndrome (IBS), postural orthostatic tachycardia syndrome (POTS), RLS, or urticaria.

All participants had the same exclusion criteria: age less than 18 or greater than 85, and inability to read English at 8th grade level or above. Subjects were also excluded if they were receiving chemotherapy, currently pregnant, or infected with mononucleosis which could all cause MCAS symptoms. Vulnerable populations were excluded.

### 2.3 Study design and methods

Review of patient records to confirm the diagnosis of MCAS was performed by each investigator. These patients received invitations to join the study by each investigator. The invitation stated that patients were being recruited to compare symptoms and conditions of MCAS versus a healthy population and that the survey was anonymous. The patients and investigators were asked to recruit their own healthy social network to the control group. The study design was observational and included retrospective and current data. To access the online questionnaire, the consent form was reviewed and affirmed before survey questions could be reviewed. Survey responses from subjects who completed less than 85 % of the survey were excluded from analysis. When disorders were not well known by their common name such as depression or anxiety, a lay definition was provided. Neurologic examples include brain fog (disturbance of memory, word-finding difficulties, difficulties in concentrating), acoustic startle (muscular activity in response to a sudden loud sound), restless legs syndrome (the compelling urge to move one's legs or arms while at rest, often associated with discomfort, usually at night and at rest, temporarily improves with movement; it is not muscular cramps or caused by pain in the back), and tinnitus (ringing or other noises in the ear(s) are bothersome and not caused by loud noise injury and do not sound like pulsations). A psychiatric example included agoraphobia: fear and avoiding places or situations that might cause panic and feelings of being trapped, helpless, or embarrassed). See Appendix 1 for other questionnaire details. In contrast, a variety of medical terms are used in this text in response to the answers. For example, the question on the survey was “do you have pain, tingling, and/or numbness in the legs or arms,” while in this text we use the medical term neuropathy.

The MC mediator release syndrome (MCMRS) questionnaire was used as a template for part of the health survey. The MCMRS score is a standardized validated checklist (Appendix 2) (Molderings et al., 2013). The updated MCMRS includes severity scores which were used to calculate the cumulative severity score (Weinstock et al., 2020-b).

### 2.4 Analysis of study results

Data included subject age, gender, ethnicity, and comparison of personal and familial health disorders. The primary outcome was the prevalence of NP disorders in MCAS subjects compared to controls. The control group with and without MCAS risk factors were compared separately to the MCAS group.

To evaluate fatigue, subjects were asked to think back to the worst part of their lifetime regarding fatigue and/or weakness and use the following scales to rate the symptoms: 1) frequency: 0 = none of the time; 1 = a little of the time; 2 = about half of the time; 3 = most of the time; 4 = all of the time; and 2) severity: 0 = symptom not present; 1 = mild; 2 = moderate; 3 = severe; 4 = very severe. The worst possible score is 8 points.

The DePaul Symptom Questionnaire was used to assess post-exertional malaise (Cotler et al., 2018) with 5 questions based on the presence or absence of five symptoms: 1) dead, heavy feeling after starting to exercise?; 2) next day soreness or fatigue after non-strenuous, everyday activities?; 3) mentally tired after the slightest effort?; 4) minimum exercise makes you physically tired?; and 5) physically drained or sick after mild activity? The worst possible score is 5 points.

The prevalence of depression was determined by asking if there was a physician's diagnosis and/or self-diagnosis of depression independent of a consequence of complications of COVID Infection. Subjects were asked: 1) if they ever had a period of depressed mood lasting most of the day almost every day for a period of at least 2 weeks; 2) if they ever had a period of at least 2 weeks in which they were much less interested in the things they usually liked to do; 3) if they ever felt deeply sad for 2 or more weeks; and 4) if they ever felt suicidal. To look for confounding causes of depression or anxiety, subjects were asked if they ever experienced abuse, trauma, or post-traumatic stress disorder (PTSD) in their lifetime.

Participants with MCAS were asked to record the historical efficacy and side effects of MC therapy by antihistamines, LDN, and benzodiazepines. Each medicine was evaluated by the percentage of the MCAS subjects who were using such medications presently or had used them in the past. Global health improvement using a Likert scale with mean (±SD) degree of improvement was based on a Likert scale of 0 (no improvement) to 10 (excellent improvement). Participants with MCAS also marked which NP symptoms improved to any degree, if any, by each medication.

### 2.5 Statistical methods

Descriptive statistics included measures of central tendency and variability for continuous variables, and percentages for categorical variables. Welch's t-tests and Chi-square tests were used to test significant differences between patients and controls on continuous and categorical variables, respectively. To compare the prevalence of NP disorders between MCAS patients and the non-age-matched controls, we performed logistic regression analyses for each disorder, separately for males and females. In each model, the presence of the NP condition (present vs. absent) was regressed on MCAS status (MCAS vs. control) while adjusting for age as a covariate. Odds ratios (OR) and 95 % confidence intervals (CI) were then computed from the regression coefficients to quantify the association between MCAS and each NP condition. Statistical analyses were performed using R version 4.41 (R Core Team, 2024). Statistical significance was defined as p < .05.

## 3 Results

### 3.1 Clinical characteristics of subjects

553 MCAS patients and 558 controls completed the survey. Patients included 499 females with mean age ±SD 45.9 ± 14.3 and 54 males with mean age 46.6 ± 15.5. Controls included 416 females with mean age 51.8 ± 15.0 and 142 males with mean age 54.1 ± 16.1. Among both genders, patients were significantly younger than controls (females p < .0001 and males p = .0036). Table 1 shows the demographics of the participants which were similar. The MCAS patients’ mean time from diagnosis of MCAS to the time of the survey was 3.0 ± 2.5 years for females and 4.3 ± 2.6 years for males.

**Table 1:** Demographics of the mast cell activation syndrome and control participants.

	Female Patients N = 499	Female Controls N = 416	Male Patients N = 54	Male Controls N = 142
Age, mean (SD)	45.9 (14.3)	51.8 (15.0)	46.6 (15.5)	54.1 (16.1)
Ethnicity, n (%)				
Caucasian	451 (90.4)	383 (91.6)	45 (83.3)	128 (90.1)
Black/African American	1	6	2	3
Hispanic/Latino	5	6	1	3
Asian	3	5	1	4
Native American or Alaska Native	0	1	0	1
Native Hawaiian or Other Pacific Islander<	0	0	2	1
Middle Eastern or North African	3	2	0	0
Other ethnicity	2	2	0	1
Multiple ethnicities	28	13	4	2
Preferred not to say	6	0	1	1

**Fig. 1:**
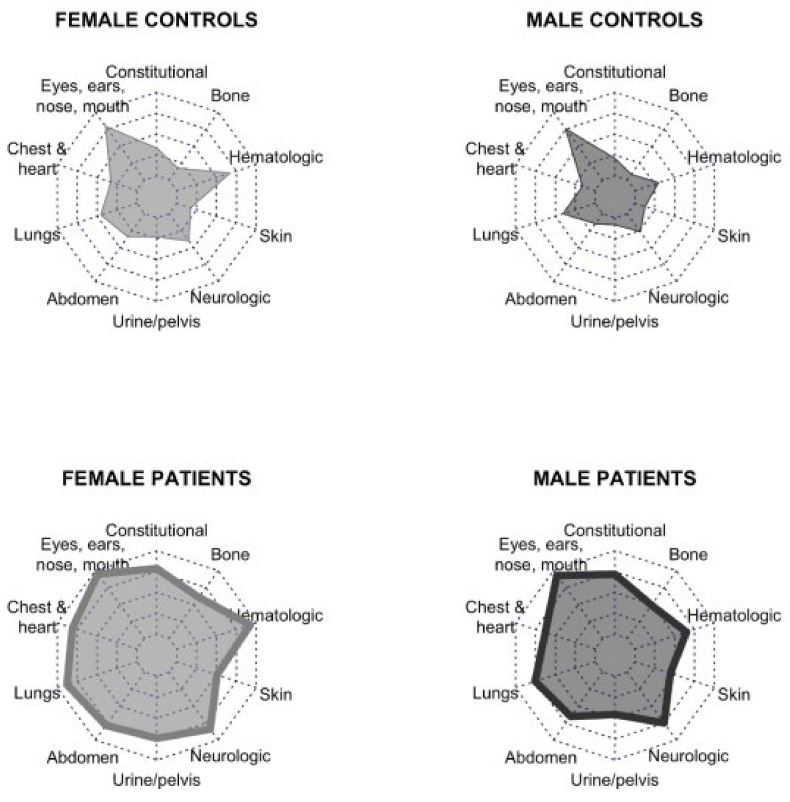
Mast cell mediator release syndrome score spiderweb plots by group and gender.

Despite their younger age, patients had accumulated all clinical risk factors compared to controls, as shown in Table 2. Patients reported significantly higher rates of MCAS-related diagnoses and family history of the diagnoses. The most common physician-diagnosed comorbid diagnoses were POTS, irritable bowel syndrome (IBS), and urticaria. The analysis of the control group with and without MCAS risk factors were compared separately to the MCAS group and there was no difference affecting the odds ratio. As shown in Table 3, patients had significantly higher overall MCMRS scores, more symptoms (i.e., symptom count), more systems involved (i.e., system count), and higher symptom severity scores compared to their same-gender controls. Spider web plots representing total MCMRS scores and system involvement in MCAS patients and control subjects are shown in Fig. 1.

**Table 2:** Participant and family clinical characteristics.

Self-reported diagnoses by physicians, n (%)	Female Patients	Female Controls	Male Patients	Male Controls
Postural orthostatic tachycardia syndrome	253 (50.1)^a^	17 (4.1)	9 (16.1)^a^	2 (1.4)
Irritable bowel syndrome	240 (47.5)^a^	71 (17.1)	27 (50)^a^	11 (7.7)
Urticaria	209 (41.4)^a^	13 (3.1)	6 (10.7)^a^	2 (1.4)
Severe allergy/asthma	191 (37.8)^a^	21 (5.0)	18 (32.1)^a^	3 (2.1)
Chronic fatigue syndrome	163 (32.3)^a^	16 (3.8)	16 (28.6)^a^	2 (1.2)
Anaphylaxis	147 (29.1)^a^	14 (3.4)	6 (10.7)	4 (2.8)
Hypermobile Ehlers-Danlos syndrome	120 (23.8)^a^	4 (1.0)	6 (10.7)^b^	1 (0.7)
Pelvic pain syndrome	114 (22.6)^a^	8 (1.9)	2 (3.6)	3 (2.1)
Cyclic vomiting syndrome	20 (4.0)^a^	1 (0.2)	1 (1.8)	1 (0.7)
Mastocytosis	16 (3.2)^b^	1 (0.2)	0	0
Participant has any of the above	456 (91.4)^a^	120 (28.8)	47 (87.0)^a^	20 (14.1)
Family member(s) had any of the above	295 (59.1)^a^	141 (33.9)	27 (50.0)^a^	40 (28.17)
Either participant or family member(s) had any of the above	471 (92.5)^a^	197 (47.4)	51 (89.5)^a^	46 (32.2)
Subject experienced physical or psychological trauma	391 (78.4)^a^	196 (47.1)	27 (50.0)	47 (33.1)
Subject experienced abuse	275 (55.1)^a^	125 (30.0)	12 (22.2)	25 (17.6)
Subject had suicidal thoughts in lifetime	274 (54.9)^a^	87 (20.9)	21 (38.9)^c^	28 (19.7)
Subject had psychiatric diagnosis or diagnoses from physician(s)	316 (63.3)^a^	129 (31.0)	26 (48.1)^a^	32 (22.5)
Subject used psychiatric medication(s)	280 (56.1)^a^	115 (27.6)	24 (44.4)^a^	25 (17.6)
Subject had ever experienced depression	437 (85.2)^a^	262 (63.0)	42 (73.7)^b^	71 (49.3)
Subject, in their lifetime, had significant fatigue and/or weakness	450 (87.7)^a^	124 (29.8)	35 (61.4)^a^	23 (16.0)
Among those who reported significant fatigue, Fatigue severity/frequency rating (0–8), Mean (SD)	6.5 (1.4)^a^	5.3 (1.6)	5.9 (1.6)^c^	5.2 (2.2)
Among those who reported significant fatigue, DePaul post-exertional malaise score (0–5), Mean (SD)	3.7 (1.6)^a^	1.1 (1.6)	2.6 (1.9)^a^	0.6 (1.3)

a MCAS patients significantly higher than same-gender controls at p < .001.

b MCAS patients significantly higher than same-gender controls at p < .01.

c MCAS patients significantly higher than same-gender controls at p < .05.

**Table 3:** Severity of symptoms of the mast cell activation syndrome participants compared to the control participants.

	Female Patients	Female Controls	Male Patients	Male Controls
MCMRS overall scores, mean (SD)^b^	30.0 (8.9)^a^	12.6 (9.6)	25.0 (10.4)^a^	7.8 (7.4)
Symptom count	25.7 (7.7)^a^	11.0 (8.6)	22.1 (9.1)^a^	7.0 (6.8)
System count	8.9 (9.9)^a^	5.6 (2.9)	7.8 (2.3)^a^	3.9 (2.7)
Severity score	162.6 (71.0)^a^	42.8 (52.3)	120.2 (68.6)^a^	22.3 (33.7)

a MCAS patients significantly higher than same-gender controls at p < .001.

b MCMRS, mast cell mediator release syndrome. SD, standard deviation.

### 3.2 Prevalence of neurologic disorders and symptoms

Female patients had a mean ± SD of 10.0 ± 4.0 self-reported neurologic disorders and symptoms, significantly higher than female controls (mean 2.5 ± 3.3, p < .001). Male patients had a mean of 7.0 ± 4.2 self-reported neurologic disorders and symptoms, significantly higher than male controls (mean 0.5 ± 2.5, p < .001). Table 4a, Table 4ba and 4b illustrate self-reported prevalence of each neurologic disorder by group and gender. The most prevalent were fatigue, cognitive dysfunction, neuropathy, and migraines, with female patients' prevalence exceeding 70 % and male patients trailing closely behind. To better characterize the major differences in prevalences between patients and controls, female and male MCAS patients' OR for each disorder were calculated, controlling for age and gender. This allowed us to quantify how MCAS was associated with (often dramatically) increased odds of having each disorder relative to the baseline rates in our control group. Female patients' OR were highest at 23.5 for cognitive dysfunction, 22.3 for fatigue, and 15.3 for non-epileptic seizure activity. Male MCAS patients’ OR were highest at 28.0 for odor sensitivity, 23.4 for light sensitivity, and 18.6 for fatigue. In other words, MCAS patients were dramatically more likely to report many neurologic disorders, suggesting the observed difference between patients and controls was not due to chance.

**Table 4a:** Female prevalences and odds ratios for neurologic disorders and symptoms.

	Prevalence in female patients	Prevalence in female controls	Odds ratio (95 % CI) for female MCAS patients a
Cognitive dysfunction	88.3 %	23.3 %	23.5 (16.6–33.7), p < .001
Chronic fatigue	86.7 %	21.6 %	22.3 (15.9–31.9), p < .001
Neuropathy	73.1 %	19.5 %	12.1 (8.8–16.8), p < .001
Migraines or severe headaches	70.0 %	27.9 %	5.6 (4.2–7.5), p < .001
Odor hypersensitivity	69.0 %	13.5 %	13.8 (9.9–19.6), p < .001
Myalgia	68.6 %	14.7 %	13.2 (9.5–18.6), p < .001
Light hypersensitivity	66.3 %	13.5 %	12.5 (8.9–17.7), p < .001
Tinnitus	59.1 %	17.5 %	7.4 (5.4–10.3), p < .001
Fainting or near faint	59.1 %	10.8 %	11.0 (7.8–15.9), p < .001
Insomnia (severe and chronic)	58.7 %	24.0 %	4.8 (3.6–6.4), p < .001
Muscle weakness	50.3 %	10.3 %	9.4 (6.5–13.7), p < .001
Sound hypersensitivity	47.4 %	11.1 %	6.9 (4.8–9.9), p < .001
Acoustic startle	44.8 %	13.0 %	5.5 (4.0–7.8), p < .001
Sleep attacks	41.1 %	13.0 %	10.0 (6.6–15.8), p < .001
Restless legs syndrome	37.2 %	11.8 %	4.4 (3.1–6.3), p < .001
Pain hypersensitivity	31.8 %	6.0 %	7.2 (4.7–11.6), p < .001
Tremors at rest	19.1 %	2.2 %	9.9 (5.2–21.3), p < .001
Non-epileptic seizure activity	16.4 %	1.2 %	15.3 (6.8–44.0), p < .001
Tourette's syndrome	3.1 %	0.0 %	N/A^b^

a CI, Confidence Intervals controlling for age.

b N/A not applicable, due to inability to calculate OR with 0 prevalence for controls.

**Table 4b:** Male prevalences and odds ratios for neurologic disorders and symptoms.

	Prevalence in male patients	Prevalence in male controls	Odds ratio (95 % CI) for male MCAS patients^a^
Cognitive dysfunction	71.9 %	16.7 %	11.7 (5.7–25.0), p < .001
Fatigue	64.9 %	8.3 %	18.6 (8.5–43.5), p < .001
Neuropathy	57.9 %	12.5 %	9.0 (4.4–19.2), p < .001
Migraines or severe headaches	47.4 %	10.4 %	7.2 (3.4–15.8), p < .001
Odor hypersensitivity	40.4 %	2.1 %	28.0 (8.9–124.4), p < .001
Myalgia	54.4 %	8.3 %	12.0 (5.5–27.4), p < .001
Light Hypersensitivity	42.1 %	2.8 %	23.4 (8.3–84.5), p < .001
Tinnitus	47.4 %	16.7 %	4.7 (2.3–9.6), p < .001
Fainting or near faint	22.8 %	4.2 %	5.8 (2.1–17.5), p < .001
Insomnia (severe and chronic)	42.1 %	9.0 %	7.7 (3.5–17.8), p < .001
Muscle weakness	33.3 %	4.2 %	10.9 (4.2–32.0), p < .001
Sound hypersensitivity	29.8 %	5.6 %	6.5 (2.6–17.1), p < .001
Acoustic startle	19.3 %	6.2 %	3.1 (1.2–8.4), p < .02
Sleep attacks	24.6 %	4.9 %	6.1 (2.3–17.2), p < .001
Restless legs syndrome	33.3 %	13.9 %	3.0 (1.4–6.4), p = .003
Pain hypersensitivity	29.8 %	4.2 %	8.8 (3.4–26.1), p < .001
Tremors at rest	15.8 %	2.8 %	7.7 (2.3–30.6), p = .002
Non-epileptic seizure activity	1.8 %	2.1 %	0.61 (0.03–5.0), p = .7
Tourette's Syndrome	1.8 %	1.4 %	0.76 (0.03–8.5), p = .8

### 3.3 Prevalence of psychiatric disorders and symptoms

Table 5a, Table 5ba and 5b illustrate self-reported prevalence of each psychiatric disorder and symptom by group and gender. The most prevalent symptoms were anxiety disorder, depression, panic attacks, and PTSD where female patients’ prevalence neared (or exceeded) 50 % and male patients trailed but still showed high prevalence compared to controls. Female patients had a mean of 3.8 ± 2.6 self-reported psychiatric disorders, significantly higher than female controls (mean 1.3 ± 1.8, p < .001). Male patients had a mean of 2.9 ± 2.7 self-reported psychiatric disorders, significantly higher than male controls (mean 0.9 ± 1.7, p < .001).

**Table 5a:** Female prevalences and odds ratios for psychiatric disorders and symptoms.

	Prevalence in female patients	Prevalence in female controls	Odds ratio (95 % CI) for female MCAS patients a
Anxiety disorder	65.9 %	30.3 %	4.0 (3.0–5.3), p < .001
Depression disorder	58.7 %	30.3 %	3.0 (2.3–4.0), p < .001
Panic disorder	48.7 %	20.0 %	3.4 (2.5–4.6), p < .001
Post traumatic stress disorder	48.3 %	12.0 %	6.6 (4.7–9.4), p < .001
Suicidal thoughts	33.9 %	6.7 %	7.4 (4.9–11.6), p < .001
Obsessive compulsive disorder	25.5 %	4.8 %	6.1 (3.8–10.3), p < .001
Agoraphobia	21.2 %	5.8 %	4.5 (2.8–7.3), p < .001
Attention deficit hyperactivity disorder	20.5 %	6.7 %	3.0 (1.9–4.8), p < .001
Anger management disorder	16.4 %	4.8 %	3.8 (2.3–6.5), p < .001
Eating disorder	17.7 %	8.7 %	2.1 (1.4–3.1), p < .001
Hallucinations	6.2 %	0.5 %	12.3 (3.4–76.6), p < .001
Mania, hypomania	7.8 %	1.9 %	3.6 (1.7–8.5), p < .001
Bipolar depression	5.8 %	0.7 %	8.1 (2.8–34.3), p < .001
Psychosis, schizophrenia	1.4 %	0.2 %	4.4 (0.77–83.3), p = .168

a Confidence Intervals (CI) controlling for age.

**Table 5b:** Male prevalences and odds ratios for psychiatric disorders and symptoms.

	Prevalence in male patients	Prevalence in male controls	Odds ratio (95 % CI) for male MCAS patients a
Anxiety disorder	49.1 %	20.8 %	3.2 (1.6–6.3), p < .001
Depression disorder	54.4 %	20.1 %	4.1 (2.1–8.2), p < .001
Panic disorder	33.3 %	12.4 %%	2.9 (1.4–6.3), p < .001
Post traumatic stress disorder	26.3 %	4.2 %	8.3 (3.1–25.2), p < .001
Suicidal thoughts	19.3 %	3.5 %	5.6 (1.9–18.9), p = .003
Obsessive compulsive disorder	31.6 %	4.9 %	8.0 (3.2–22.2), p < .001
Agoraphobia	24.6 %	4.9 %	7.94 (2.9–23.5), p < .001
Attention deficit hyperactivity disorder	14.0 %	6.9 %	1.7 (0.6–4.6), p = .3
Anger management disorder	22.8 %	0.9 %	2.6 (1.1–6.2), p = .03
Eating disorder	7.0 %	2.1 %	2.9 (0.6–15.5), p = .2
Hallucinations	5.3 %	0.7 %	7.5 (0.9–156.2), p = .1
Mania, hypomania	5.3 %	2.1 %	2.3 (0.4–13.4), p = .3
Bipolar depression	3.5 %	2.8 %	1.2 (0.1–6.4), p = .9
Psychosis, schizophrenia	1.8 %	0.7 %	1.57 (0.1–42.1), p = .7

a Confidence Intervals (CI) controlling for age.

To better characterize the significant differences in prevalences between patients and controls, the OR was calculated for each symptom, separately for males and females, and controlled for age. Female patients' OR were statistically significantly higher than female controls on 13 of 14 disorders, and highest at OR 12.3 for hallucinations, 8.1 for bipolar depression, 7.4 for suicidal ideation, 6.6 for PTSD and 6.1 for OCD. Male patients’ OR were statistically significantly higher than male controls on 8 of 14 disorders, and highest at OR 8.3 for PTSD, 8.0 for OCD, 8.3, 7.9 for agoraphobia, and 7.0 for hallucinations.

### 3.4 Response to medical therapy for both neurologic and psychiatric disorders and symptoms

The number and percent of MCAS subjects who tried antihistamines was 498 (88.9 %), LDN was 347 (62.0 %), and benzodiazepines was 324 (57. 9 %). In self-reported ratings for effects on health status (0 = no benefit, 10 = maximum conceivable benefit), MCAS patients gave slightly, but significantly higher mean ratings (±SD) to antihistamines 6.3 (±2.3) vs. LDN 5.6 (±3.2) and benzodiazepines 5.6 (±3.1) (p < .01).

In the subjects who experienced the following neurologic disorders and symptoms, antihistamines were rated effective by 25 % of the patients who used antihistamines for cognitive dysfunction, 23 % for migraine, 22 % for fatigue, and 21 % for chronic severe insomnia. LDN was rated to be effective by 29 % for fatigue and myalgia, 28 % for cognitive dysfunction, and 22 % for both neuropathy and RLS. Benzodiazepines helped 51 % of those with chronic severe insomnia, 34 % with tremors at rest, 29 % with RLS, 25 % with pain hypersensitivity, and 22 % with myalgia. Further details are shown in Table 6a.

**Table 6a:** Number (%) of MCAS patients reporting that antihistamines, low-dose naltrexone, or benzodiazepines helped their neurologic disorders or symptoms. a

Condition	Antihistamines	Low-dose Naltrexone	Benzodiazepines
Fatigue	94/434 (21.7 %)	91/309 (29.4 %)	45/294 (15.3 %)
Cognitive dysfunction	113/445 (25.4 %)	88/315 (27.9 %)	63/300 (21.0 %)
Migraine-like headaches	82/352 (23.3 %)	25/244 (10.2 %)	52/243 (21.4 %)
Faint or near faint	42/286 (14.7 %)	18/197 (9.1 %)	25/206 (12.1 %)
Insomnia: chronic/severe	63/299 (21.1 %)	29/204 (14.2 %)	110/217 (50.7 %)
Odor hypersensitivity	69/342 (20.2 %)	18/234 (7.7 %)	33/235 (14.0 %)
Pain hypersensitivity	25/159 (15.7 %)	21/124 (16.9 %)	30/122 (24.6 %)
Light hypersensitivity	54/335 (16.1 %)	20/230 (8.7 %)	24/233 (10.3 %)
Sound hypersensitivity	18/230 (7.8 %)	13/168 (7.7 %)	31/166 (18.7 %)
Tinnitus	37/295 (12.5 %)	20/202 (9.9 %)	19/200 (9.5 %)
Myalgia	54/342 (15.8 %)	73/252 (29.0 %)	54/242 (22.3 %)
Muscle weakness	25/245 (10.2 %)	19/172 (11.0 %)	16/185 (8.6 %)
Neuropathy	51/368 (13.9 %)	57/258 (22.1 %)	53/250 (21.2 %)
Tremor at rest	14/98 (14.3 %)	5/69 (7.2 %)	27/79 (34.2 %)
Restless legs syndrome	23/186 (12.4 %)	31/139 (22.3 %)	40/136 (29.4 %)
Dystonia	8/81 (9.9 %)	3/46 (6.5 %)	12/63 (19.0 %)
Sleep attacks	22/203 (10.8 %)	17/144 (11.8 %)	9/149 (6.0 %)
Acoustic startle	6/218 (2.8 %)	4/158 (2.5 %)	29/170 (17.1 %)
Non-epileptic seizure activity	9/79 (11.4 %)	1/46 (2.2 %)	17/61 (27.9 %)
Tourette's syndrome	2/16 (12.5 %)	2/12 (16.7 %)	2/12 (16.7 %)

Data was not collected to assess whether patients were taking these medicines alone or simultaneously.

a The numerator is number of patients reporting the medication that helped the disorder. Denominator is number of patients with the disorder who took the medication.

In the subjects who experienced the following psychiatric disorders and symptoms, antihistamines were rated effective by 21 % for anxiety disorder, 14 % for anger management problems, 13 % for depression, and 12 % for panic disorder. LDN was rated effective by 16 % for anxiety disorder and depression, 14 % for bipolar disorder, 12 % for agoraphobia, and 10 % for ADHD. Benzodiazepines showed the highest efficacy ratings for anxiety disorders (helping 70 %), panic disorder (52 %), agoraphobia (51 %), OCD (38 %), PTSD (33 %), and anger management (32 %). Among 324 patients who took benzodiazepines, 76 (23.4 %) had to increase dose to maintain the same effect. Further details are shown in Table 6b. Specific symptoms were summarized in supplemental files 1 to 3.

**Table 6b:** Number (%) of MCAS patients reporting that antihistamines, low-dose naltrexone, or benzodiazepines helped their psychiatric disorders or symptoms. a

Condition	Antihistamines	Low-dose Naltrexone	Benzodiazepines
Anxiety disorder	70/326 (21.5 %)	36/229 (15.7 %)	172/245 (70.2 %)
Agoraphobia	10/110 (9.1 %)	8/65 (12.3 %)	45/89 (50.6 %)
Panic disorder	29/240 (12.1 %)	9/169 (5.3 %)	99/189 (52.4 %)
Anger management problem	12/88 (13.6 %)	3/65 (4.6 %)	24/75 (32.0 %)
Depression	37/297 (12.5 %)	36/220 (16.4 %)	65/223 (29.1 %)
Bipolar depression	3/25 (12.0 %)	3/22 (13.6 %)	5/26 (19.2 %)
Mania, hypomania	4/36 (11.1 %)	2/26 (7.7 %)	4/34 (11.8 %)
Suicidal thoughts	0/170 (0.0 %)	0/120 (0.0 %)	0/121 (0.0 %)
Psychosis, schizophrenia	0/8 (0.0 %)	0/6 (0.0 %)	2/7 (28.6 %)
Hallucinations	0/31 (0.0 %)	1/19 (5.3 %)	4/26 (15.4 %)
Eating disorders	0/85 (0.0 %)	0/66 (0.0 %)	0/69 (0.0 %)
Obsessive compulsive disorder	9/133 (6.8 %)	5/98 (5.1 %)	35/105 (33.3 %)
Attention deficit hyperactivity disorder	9/106 (8.5 %)	8/82 (9.8 %)	9/83 (9.6 %)
Post-traumatic stress disorder	15/238 (6.3 %)	8/156 (5.1 %)	70/181 (38.7 %)

a Numerator is number of patients reporting that the medication helped the disorder. Denominator is the number of patients with the disorder who took the medication. The denominator is different for every medication efficacy number, because not every patient tried every medication and not every patient had every symptom/disorder.

### 3.5 Side effects of medical therapy for neuropsychiatric disorders

Side effects were reported by patients at a rate of 38.5 % for antihistamines, 33.7 % for LDN, and 34.9 % for benzodiazepines, with further details shown in Table 7. Among 324 patients who took benzodiazepines, 76 (23.4 %) had to increase the dose to maintain the same effect, yet specific data about whether this drug class was used as monotherapy was not assessed in the questionnaire. Patients reported a wide range of side effects to these medications, though it is unclear whether such issues were drug-driven reactivities or, as MCAS is known to drive, excipient-driven reactivities (Schofield, Afrin, 2019).

**Table 7:** Overall self-rated side effects of antihistamines, low-dose naltrexone, and benzodiazepines.

	Antihistamines	Low-dose Naltrexone	Benzodiazepines
Tried the medication, n (%)	498 (88.9)	347 (62.0)	324 (57.9)
Experienced side effects	192 (38.5)	117 (33.7)	113 (34.9)
Side effects resolved and medicine was continued	41 (21.3)	34 (29.0)	Not queried
Side effects led to cessation of the medication	81 (42.2)	69 (59.0)	70 (61.9)

Well-established general physiological effects (dryness of mucus membranes – 12 patients) and neurological and neuropsychiatric effects (fatigue – 9 patients) were common for those who tried type-1 antihistamines. The use of sedating versus nonsedating antihistamines was not questioned in the survey (Appendix 1). LDN was associated with a large variety of side effects including headaches, insomnia, and abnormal dreams. The most reported side effects from benzodiazepines were drowsiness (22 patients), fatigue (19 patients), brain fog (18 patients), and other memory issues (15 patients). Increased anxiety in 10 patients and worsening depression in 8 patients was also reported. Fourteen patients reported anaphylaxis and hives from benzodiazepines.

## 4 Discussion

### 4.1 Overview

The present study showed that MCAS patients have high rates of self-reported NP disorders and symptoms compared to healthy age-matched controls. For neurologic disorders, the prevalence in descending order from 75 % to 25 % included fatigue, cognitive dysfunction, migraine-like headaches, faint/near faint, insomnia, environmental sensitivities (odor, light, sound), tinnitus, muscle tenderness, neuropathy-like symptoms, muscle weakness, RLS, pain hypersensitivity, and acoustic startle. For psychiatric disorders, the highest prevalences observed in descending order from 75 % to 25 % included anxiety, depression, panic disorder, PTSD, suicidal thoughts, and OCD.

We theorize that, when inappropriately expressed, MC mediators which either cross the BBB into the CNS or which are expressed by MCs resident within the CNS can lead to or exacerbate various NP disorders. Certain MC mediators, specific genetic predisposition, and life experiences could determine which disorder is apt to develop or worsen. NP disorders could also be affected by genetically mutated MCs in the CNS and/or peripheral nervous system or by circulating MC mediators that may lead to CNS or peripheral nervous system neuroinflammation.

Although histamine itself does not readily cross the blood-brain barrier (BBB) (Partridge, 2005), histamine generated by any cells within the CNS (including at least some neurons as well as MCs, macrophages, and some glial cells) obviously has potential to engage with any cells within the CNS which express histamine receptors. Most of the histamine receptors (H1, H2, and H4 (Haas et al., 2008; Szukiewicz, 2024), found on most MCs are also found on most neurons (evidence is weaker regarding neuronal expression of H4 receptors (Schneider, Seifert, 2016), so it is inescapable that aberrantly excessive histamine expression by the fundamentally dysfunctional MCs at the root of MCAS (and regardless of whether such MCs are resident within the CNS or outside) has ample potential to drive a wide array of problems in both peripheral and central neurons both directly via binding with neuronal histamine receptors as well as indirectly by binding with various immune and glial cells in the central nervous system. Depending on which mediators are (directly and/or indirectly) impacting which neurons, such effects obviously can result in any of a wide array of central neuronal issues (Szukiewicz, 2024) regardless of whether any such issue is superficially categorized as a “neurologic” or “cognitive” or “psychiatric” or “dysautonomic” issue. A similarly wide array of direct and indirect effects upon neurons can be seen with many other potent inflammatory mediators, too, which are expressed by MCs (some of which, unlike histamine, readily cross the BBB (e.g., interleukin-6 (IL-6) and tumor necrosis factor alpha (TNF-alpha) (Banks, 1995). Though the BBB penetrability of another potent inflammatory mediator produced by MCs and glial cells, IL-12 is less clear. IL-12 is clearly involved in pathogenesis and progression of a variety of neuropsychiatric disorders (Chauhan, 2025).

MCAS is often unrecognized and undiagnosed (Afrin et al., 2016-b). This multisystemic disease can account for the underlying pathophysiology in certain idiopathic syndromes including POTS, FMS, CPPS, and IBS. These syndromes have been labeled as have underlying centralized hypersensitivity. Physicians should consider trying to diagnose MCAS and using MCAS therapies as part of the approach to the complex patient who might otherwise be dismissed as having a functional neurologic or somatoform disorder.

A high burden of NP manifestations has also been documented in POTS, hEDS, and fibromyalgia. In an online survey of 4835 individuals with POTS, 99 % reported dizziness or light-headedness, 94 % difficulty concentrating, 94 % headache, 87 % memory problems, 84 % myalgia, 83 % muscle weakness, 78 % tremulousness, 76 % paresthesias in the hands and 67 % in the feet, and 75 % blurred vision, 65 % hand numbness, 58 % foot numbness (Shaw et al., 2019). After POTS diagnosis, 37 % reported having a psychiatric or psychological diagnosis. Likewise, an online survey of 2596 people with fibromyalgia found that 47 % reported recurrent headaches, 46 % tingling, 45 % balance problems, 44 % numbness, 40 % chronic fatigue, 40 % depression, 38 % anxiety and 30 % tinnitus (Bennett et al., 2007). A review of neurological symptoms in hEDS identified pain, fatigue, and headache as leading reasons for seeking care and deterioration of quality of life (Castori and Voermans, 2014).

### 4.2 Comments on specific neurological disorders and symptoms

In the present study, fatigue and cognitive dysfunction had the highest OR (20.5 and 20.1, respectively). LDN was rated helpful by 29 % for fatigue and antihistamines were rated helpful in 22 % of patients who took LDN. LDN helped 28 % with cognitive dysfunction and antihistamines helped 22 %. Based on limitations of the questionnaire, it is not clear whether these benefits were additive using multiple medications.

POTS can be a devastating multi-systemic illness and can have comorbid MCAS (Kohno et al., 2021). In a study of 69 POTS patients, 42 % (29/69) initially diagnosed with POTS showed both additional symptoms and at least one elevated biochemical marker suggesting MCAS (Kohno et al., 2021). Another study of the parasympathetic nervous system and MCs suggested that endogenous acetylcholine (receptors for which are expressed by MCs) activates meningeal MCs (Kilinc et al., 2024). Further studies are needed to delineate the complex interplay between MCs, the autonomic nervous system, connective tissues of the meninges, cerebral vasculature, and other structures important to the pathophysiology of the triad of dysautonomia, MCAS, and hypermobility spectrum disorders including hypermobile Ehlers-Danlos syndrome (Wang et al., 2021).

In the present study, POTS occurred in 50.1 % of MCAS females vs. 4.1 % of control females. Studies of comorbid POTS in MCAS patients are limited. One study showed the prevalence of POTS in 24.7 % of 174 MCAS patients (Weinstock et al., 2020b). Patients with both syndromes are often more ill than patients with one disorder alone (Weinstock et al., 2021a, Weinstock et al., 2021b, Weinstock et al., 2021c). Prevalence differences could also be explained if more severely affected MCAS participants with co-morbid POTS were more inclined to take the questionnaire study. The other reason could be that the present study was a multicenter study whereas the other study recruited patients from refractory gastrointestinal patients.

FMS is a common idiopathic disorder marked by chronic widespread pain along with myalgia, muscle sensitivity, fatigue, and insomnia and is associated with migraines, atopic disorders, and depression (Fitzcharles et al., 2021; Tsiakir et al., 2017; Yepez et al., 2022). It is thought that FMS is primarily caused by central hypersensitivity (Berwick et al., 2022), yet inflammation with cytokines produced by MCs has also been discovered to play a role and cutaneous MC counts are increased in FMS (Parkitny, Younger, 2017; Enestrom et al., 1997). All signs and symptoms of FMS overlap symptoms of MCAS, and both can start at an early age (Afrin et al., 2015, 2017; Berwick et al., 2022; Coles et al., 2021).

RLS occurs in 7–10 % of the general population, increases morbidity and mortality, and is associated with immune, inflammatory, and infectious causes in 42 highly associated disorders with secondary RLS (Li et al., 2018; Weinstock et al., 2012). In the present study, 37.2 % of females had RLS (OR 4.4) and 33.3 % of men had RLS (OR 3.0). In a prior study, 174 MCAS patients (146 female, 28 males, mean age 44.8 years) were compared to 85 spouse controls (12 females, 73 males, mean age 50.9 years). Female MCAS patients had a higher prevalence of RLS (40.8 %) than spouse controls (12.9 %) (p < .0001) (Weinstock et al., 2020-c). Endorphin deficiency in the brain appears to contribute to RLS and LDN could reverse this deficiency (Walters et al., 2024). In the present study, 22 % of the MCAS patients with RLS had improvement with LDN.

Tinnitus causes a poor quality of life (Batts, Stankovic, 2024). In the present study, 59.1 % of female patients had tinnitus with an OR of 7.4. Men had similar prevalence and OR (47.1 % and 4.7, respectively). An earlier study of tinnitus showed a prevalence of 61.4 % of 114 predominately female MCAS patients (Weinstock et al., 2021a, Weinstock et al., 2021b, Weinstock et al., 2021c).

### 4.3 Specific comments about benzodiazepines

We have seen that benzodiazepines are sometimes being prescribed for anxiety and insomnia before the patients were diagnosed with MCAS. We have documented that anxiety and insomnia are common disorders in MCAS. Although benzodiazepines have been widely used in patients with these NP disorders, their chronic use may be associated with side effects, including sedation, abuse, dependence, and cognitive and mood alterations in a subset of patients. In our patient population with MCAS, we have found low dose, judicious use of benzodiazepines to be helpful both in the setting of chronic MC activation and/or as a treatment for an acute flare, with minimal side effects and minimal risk for development of addiction/tolerance. Some patients find benzodiazepines to help abortively for flares, some find them helpful preventively using a low dose 1–3 times daily, and some find them helpful in both contexts.

### 4.4 Limitations of the study

This study has several limitations. 1) This was a volunteer study and thus participants have potential self-selection bias which could skew the data. The self-reported diagnoses, treatment and outcomes could not be confirmed and may have been influenced by recall bias. Confirmation of the survey responses was not possible in this computer-based anonymous survey. 2) There was a high percentage of female MCAS subjects, although this is representative of MCAS patients in the literature (and thought to be due in part to the activating effects of estrogen when engaging with MC-surface estrogen receptors) (Molderings et al., 2013; Afrin et al., 2017). 3) COVID-19 infections may have affected results by exacerbating/flaring MCAS and sparking Long COVID Syndrome (Afrin, 2020-b; Weinstock, 2021-a). We addressed this concern by asking affected participants to consider their responses to pre-COVID-19 symptomology. Health changes provoked by asymptomatic SARS-CoV-2 infections could not be determined. 4) Assessment of medication response may have been influenced by concomitant neurologic and MCAS medications. MCAS patients usually are treated with multiple medications and thus judging the efficacy of adding a new one can be difficult without double-blind prospective study or using monotherapy, which is not standard of care in clinical practice. 5) Our questionnaire did not address how many medicine classes were used and whether more than one type of antihistamine was used. A sizable percentage of MCAS patients had tried one or more benzodiazepine. Benzodiazepine-responsive disorders are common in MCAS, and benzodiazepines reduce MC activity in laboratory studies.

## 5 Conclusions

Recognition that NP disorders are highly prevalent in patients with MCAS may lead to promising therapeutic treatment options for patients with MCAS and those with NP disorders who are refractory to standard NP medications and whose MCAS diagnosis may have been missed. Treatment directed at the uncontrolled, aberrant MC can potentially improve some NP disorders and symptoms. These observations and hypotheses will need evaluation in future prospective, randomized, placebo-controlled trials.

## CRediT authorship contribution statement

**Leonard B. Weinstock:** Writing – review & editing, Writing – original draft, Supervision, Conceptualization. **Lawrence B. Afrin:** Writing – review & editing, Writing – original draft, Methodology. **Angela M. Reiersen:** Writing – review & editing, Writing – original draft, Methodology, Investigation. **Jill Brook:** Methodology, Investigation, Formal analysis, Data curation. **Svetlana Blitshteyn:** Writing – review & editing, Methodology. **Gillian Ehrlich:** Writing – review & editing, Investigation. **Jill R. Schofield:** Writing – review & editing, Investigation. **Laurence Kinsella:** Writing – review & editing, Resources. **David Kaufman:** Resources, Investigation. **Tania Dempsey:** Resources, Investigation. **Gerhard J. Molderings:** Writing – review & editing, Writing – original draft, Supervision, Methodology, Investigation, Conceptualization.

## Disclaimer statement

Dr. Molderings is the co-owner and chief medical officer in the start-up company Mast Cell Sciences Ltd. Drs. Afrin and Weinstock are uncompensated, voluntary medical advisors to Mast Cell Sciences Ltd. All other authors report no conflicts of interest. No one has competing interests.

## Support

None.

## Declaration of competing interest

Dr. Molderings is co-owner and chief medical officer in the start-up company Mast Cell Sciences Ltd. All other authors report no conflicts of interest. No one has competing interests.

## Data Availability

Data are attached as Supplementary files.
