# Supplementary material for: Prevalence and treatment response of neuropsychiatric disorders in mast cell activation syndrome
Source: Brain Behav Immun Health. 2025 Jun 30;48:101048. doi: 10.1016/j.bbih.2025.101048 (PMC12270938; doi:10.1016/j.bbih.2025.101048)
Supplement: Multimedia component 1 [file mmc1.docx]

1. Have YOU been diagnosed by a physician with any of the following conditions?  Please check all that apply:

- Anaphylaxis
- Chronic fatigue syndrome
- Chronic pelvic pain syndromes (interstitial cystitis, chronic prostatitis, vulvodynia)
- Cyclic vomiting syndrome
- Ehlers-Danlos syndrome (EDS)
- Fibromyalgia
- Hives
- Mastocytosis
- Postural orthostatic tachycardia syndrome (POTS)
- Severe allergic symptoms and/or asthma requiring medicines on a weekly basis year-round

2. Has ANYONE IN YOUR FAMILY been diagnosed with the following conditions?  Please check all that apply:

- Anaphylaxis
- Chronic fatigue syndrome
- Chronic pelvic pain syndromes (interstitial cystitis, chronic prostatitis, vulvodynia)
- Cyclic vomiting syndrome
- Hypermobile Ehlers-Danlos syndrome (EDS)
- Fibromyalgia
- Hives
- Mastocytosis
- Postural orthostatic tachycardia syndrome (POTS)
- Severe allergic symptoms and/or asthma requiring medicines on a weekly basis year-round

3. What is your age?

4. What was your sex at birth?

- Female
- Male

5. What is your ethnicity? (check all that apply)

- White
- Black or African American
- Hispanic or Latino
- Asian
- Native American or Alaska Native
- Native Hawaiian or Other Pacific Islander
- Middle Eastern or North African
- Other (please specify): ________
- Prefer not to say

6. Have you been diagnosed with - or have you believed you had - any of the following? Check all that apply and type in the approximate age that it started:

- Agoraphobia (fear and avoiding places or situations that might cause panic and feelings of being trapped, helpless, or embarrassed)
- Anger management or difficulty controlling your anger
- Anxiety disorder
- Attention deficit disorder (ADD)
- Attention deficit hyperactivity disorder (ADHD)
- Bipolar depression
- Brain fog (disturbance of memory, word-finding difficulties, difficulties in concentrating)
- Depression
- Dystonia (movement of the body that you cannot control)
- Eating disorder
- Fainting or near fainting
- Fatigue (significant, on a regular basis)
- Hallucinations
- Migraine-like headaches
- Light and/or sun sensitivity
- Mania or hypomania
- Muscle pain or tenderness
- Muscle tightness caused by hyperventilation
- Muscle weakness in the legs or arms
- Nerve pain, numbness, tingling in the legs or arms
- Obsessive compulsive disorder (OCD)
- Increased sensitivity to feeling pain and/or an extreme response to pain
- Panic attacks (these may have occurred as a teenager or child)
- Post-traumatic stress syndrome or disorder (PTSD)
- Psychosis or schizophrenia
- Restless legs syndrome (the compelling urge to move your legs or arms while at rest, often associated with discomfort, usually at night and at rest, temporarily improves with movement. It is not muscular cramps or resulting from pain in the back)
- Seizure-like activity
- Insomnia
- Irresistible drowsiness/sleep attacks
- Significant sensitivity to odors/scents (e.g., nausea, headache, or passing out)
- Loss of the ability to smell
- Ringing or other noises in the ear(s) bother you and were not caused by loud noise injury and do not sound like pulsations
- Sensitivity to noises that most people do not hear and bothers you a lot
- Acoustic startle (muscular activity in response to a sudden loud sound)
- Tourette's syndrome with verbal or movement tics (now or in the past)
- Tremor at rest
- Other neurological or psychological problems - please describe:  ___________
- None of the above

Did all of the problems above only happen after getting COVID-19? Yes/No

7. Have you had suicidal thoughts in your lifetime? Yes/No

8. Have you been given a psychiatric diagnosis by a doctor (example: depression)? Yes/No

(If no, skip 9-15)

9. If yes, type in this diagnoses: ___

10. Have you agreed with the doctor's diagnosis for one or more of the psychiatric conditions? Yes/No

11. Did you receive psychiatric medications? Yes/No

12. Did you receive other treatments (example: talk therapy)? Yes/No 
If yes, type what treatments were these? ___

13. Were the psychiatric/therapeutic interventions helpful? Yes/No

14. Please type out the names of any medications that were helpful: ___

15. Please type out the names of any medications with bad side effects: __

For all participants:

16. Have you ever experienced physical or psychological trauma in your lifetime? Yes/No

17. Have you ever experienced abuse in your lifetime? Yes/No

18. Have you ever experienced depressed (do not include complications of COVID-19 Infection) Yes/No

If never depressed, skip 19-21

19. Did you ever have a period of depressed mood lasting most of the day almost every day for a period of at least 2 weeks? Yes/No

20. Did you ever have a period of at least 2 weeks in which you were much less interested in the things you usually like to do? Yes/No

21. Have you ever felt deeply sad for 2 or more weeks? Yes/No

For all participants:

22. In your lifetime, did you have significant fatigue and/or weakness (do not include complications of COVID19 Infection)?  Yes/No

(If no, skip 23 - 26 and go to 27)

23. Think back to the worst part of your lifetime with fatigue and/or weakness and use the following scales to rate the symptoms below

24. Symptom Frequency:

0 = none of the time

1 = a little of the time

2 = about half of the time

3 = most of the time

4 = all of the time

25. Symptom Severity: During this time, how much did this symptom bother you?

0 = symptom not present

1 = mild

2 = moderate

3 = severe

4 = very severe

26. Have you had the following symptoms

- Dead, heavy feeling after starting to exercise? Yes/No
- Next day soreness or fatigue after non-strenuous, everyday activities? Yes/No
- Mentally tired after the slightest effort? Yes/No
- Minimum exercise makes you physically tired? Yes/No
- Physically drained or sick after mild activity? Yes/No

For all participants:

27. In the following section, think about your current and past medical history. If you had long term symptoms resulting from a COVID-19 infection, answer the questions the best you can thinking about your health prior to that infection.

- Did you get colds regularly which then turn into bacterial infections such as bronchitis or sinus infections? Yes/No
- Was the course of illnesses episodic (and/or with attacks)? Yes/No
- Did symptom-free periods become shorter and shorter? Yes/No

The following symptoms may have occurred repeatedly or may be constant. Please rate frequency and severity of each (0 to 10-point scale for each). Zero (0) means you never have had this problem.

28. GENERAL HEALTH

1. Significant physical weakness or fatigue doing everyday activities
2. Extreme fatigue attacks, it can be hard to keep eyes open
3. Weight loss despite maintaining my normal diet

29. EYES, EARS, NOSE, MOUTH

1. Ears have ringing or odd sounds
2. Eyes are dry, itchy, red, burning, or feel gritty
3. Runny or stuffy nose
4. Inflammation or ulcers of the mouth

30. CHEST and HEART

1. Burning and/or pressure pain in the chest (normal electrocardiogram or stress test; or not severe enough to go to ER)
2. Rapid heart rate (rapid palpitations)
3. Redness or flushing of the skin, especially face or upper body
4. Hot flashes (usually with dry skin lasting 2 to 5 minutes, rarely more than 10 minutes and often occur with nausea or other symptoms. These are not menopausal hot flashes with wet sweats)
5. Sudden dizziness/lightheadedness with fainting or near fainting

31. LUNGS

1. Irritable dry cough or need to cough
2. Feeling of shortness of breath or difficulty taking a full breath
3. Asthma-like complaints (wheezing)

32. ABDOMEN

1. Attacks of visible bloating or distention within minutes
2. Pain in the abdomen
3. Pain is burning
4. Pain is crampy or spastic
5. Pain is associated with diarrhea (watery or loose stool)
6. Nausea (with or without vomiting)
7. Do antihistamines help reduce nausea? (Allegra, cetirizine, Claritin, diphenhydramine, hydroxyzine, Ketotifen, loratadine, Xyzal, Zyrtec)

33. URINE/PELVIS

1. Bladder and/or pelvic pain (this applies to both women and men and is often associated with painful, frequent, and/or urgent urination and may be associated with sexual intercourse. During these times bacterial cultures and urine analysis are normal)

34. NEUROLOGIC and MUSCULOSKELETAL

1. Migraine-like headaches (throbbing on one side only or have been diagnosed as a migraine -- these are NOT tension headaches)
2. Brain fog -- word finding problems and/or concentration difficulties with or without associated insomnia episodes
3. Leg or arm pain and/or altered feelings including numbness, tingling, burning, sharp pain, and pins and needles (this does not respond to over-the-counter pain medicine)

35. SKIN

1. Hives (red raised itchy spots)
2. Hemangiomas (raised or flat bright red spots)
3. During attacks there are itchy skin lesions that look like acne in the corners of the nasal-lip area, as well as the chin and forehead
4. Knots or nodules under the skin
5. Painless, non-itchy swelling (especially lips, cheeks, eyelids)
6. Itching in area around the anus during attacks

36. HEMATOLOGIC

1. Bruising after minor injuries
2. Unusual nose bleeds
3. Women only: significant menstrual bleeding

37. TRIGGERS Are any of the symptoms or complaints listed above worsened by:

1. High histamine foods (such as alcohol, cheese, chocolate, tuna, cured fish/meat, left-over meat, raisins, tomatoes)
2. Sleep deprivation (awake for more than 24 hours)
3. Hunger or fasting (no food all day)

38. BONE

1. Bone pain that usually occurs in more than one bone
2. Bone density test showed osteoporosis or osteopenia without a known cause

**B. MCAS Patient Treatment Questionnaire: Benzodiazepine Therapy**

Now or in the past, did you take one or more of the following benzodiazepine medicines: Ativan (lorazepam), Valium (diazepam), Klonopin (clonazepam), and/or Xanax (alprazolam)? Yes/No

(If no, skip 1 - 8)

1. These medicines have only been taken as pills.  Yes/No
2. Some have been given intravenously. Yes/No
3. Please write in your best benzodiazepine medicine and best dose: ___
4. Rate the overall effect of benzodiazepines on your overall health status from 0 (no improvement) to 10 (excellent improvement).
5. Type in which symptoms were improved the most:___
6. If you had any of the following conditions, check the conditions that were improved by these benzodiazepine medicines:

- Agoraphobia (fear and avoiding places or situations that might cause panic and feelings of being trapped, helpless, or embarrassed)
- Anger management or difficulty controlling your anger
- Anxiety disorder
- Attention deficit disorder (ADD)
- Attention deficit hyperactivity disorder (ADHD)
- Bipolar depression
- Brain fog (disturbance of memory, word-finding difficulties, difficulties in concentrating)
- Depression
- Dystonia (movement of the body that you cannot control)
- Fainting or near fainting
- Fatigue (significant, on a regular basis)
- Hallucinations
- Migraine-like headaches
- Light and/or sun sensitivity
- Mania or hypomania
- Muscle pain or tenderness
- Muscle tightness caused by hyperventilation
- Muscle weakness in the legs or arms
- Nerve pain, numbness, tingling in the legs or arms
- Obsessive compulsive disorder (OCD)
- Increased sensitivity to feeling pain and/or an extreme response to pain
- Panic attacks (these may have occurred as a teenager or child)
- Post-traumatic stress syndrome or disorder (PTSD)
- Psychosis or schizophrenia
- Restless legs syndrome (the compelling urge to move your legs or arms while at rest, often associated with discomfort, usually at night and at rest, temporarily improves with movement. It is not muscular cramps or resulting from pain in the back)
- Seizure-like activity
- Insomnia
- Irresistible drowsiness/sleep attacks
- Significant sensitivity to odors/scents (e.g., nausea, headache, or passing out)
- Loss of the ability to smell
- Ringing or other noises in the ear(s) bother you and were not caused by loud noise injury and do not sound like pulsations
- Sensitivity to noises that most people do not hear and bothers you a lot
- Acoustic startle (muscular activity in response to a sudden loud sound)
- Tourette's syndrome with verbal or movement tics (now or in the past)
- Tremor at rest
- Other problems - please write here: ___
- None of the above: ___
- I am not sure since I was on other medicines at the time: ___

1. Did you need to increase the dose of the benzodiazepine over time to maintain the same effect?  Yes/No
2. Have any health professionals accused you of abusing or being addicted to these medicines?  Yes/No



 **C. MCAS Patient Treatment Questionnaire: Low Dose Naltrexone**

Now or in the past, were you treated with low dose naltrexone (LDN)?  Yes/No

(If No, skip 1-10):

1. If you take naltrexone now, think about how it affects your overall health status. Rate the effect from 0 (no improvement) to 10 (excellent improvement)
2. If you took naltrexone in the past, think about how it affected your overall health status. Rate the effect from 0 (no improvement) to 10 (excellent improvement):
3. What symptoms were improved the most (type in your response):___
4. If you had any of the following conditions, check the boxes of the conditions that were improved by naltrexone:

Psychologic disorders

anxiety disorder, agoraphobia, panic attacks, depression, bipolar depression, mania/hypomania, psychosis/schizophrenia, hallucinations, obsessive compulsive disorder, attention deficit hyperactivity disorder, anger management, post-traumatic stress disorder

Neurologic disorders

fatigue, brain fog (disturbance of memory, word-finding difficulties, difficulties in concentrating, cognitive dysfunction), faint/near faint, migraine-like headaches, muscle pain/tenderness, muscle tightness caused by hyperventilation, muscle weakness in the arms/legs, nerve pain/numbness/tingling, restless legs syndrome, seizure-like activity, insomnia, sleep attacks, anosmia, tinnitus, misophonia, acoustic startle, Tourette's syndrome, dystonia, resting tremor, and hypersensitivity to (a) light/sun, (b) pain, (c) odors/scents, or (d) noise.

- Other problems - please list here: ___
- None of the above: ___
- I am not sure since I was on other medicines at the time: ___

5. Did you have side effects from naltrexone?  Yes/No

6. If yes, did the side effects resolve over time while continuing the naltrexone?  Yes/No

7. Please write in side effects:___

8. Did the side effects make you stop taking it?  Yes/No

9. Were you able to continue naltrexone on a lower dose and find that it was helpful?  Yes/No/Not Applicable

10. Type in which was the best dose of naltrexone for you:___

**D. MCAS Patient Treatment Questionnaire: Antihistamines**

Now or in the past, were you treated with antihistamines? Yes/No

(If no, skip 1-8):

1. If you take antihistamines now, think about how it affects your overall health status. Rate the effect from 0 (no improvement) to 10 (excellent improvement):

2. If you took antihistamines in the past, think about how it affected your overall health status. Rate the effect from 0 (no improvement) to 10 (excellent improvement):

3. What symptoms were improved the most (type in your response):___

4. If you had any of the following conditions, check the boxes of the conditions that were improved by antihistamines:

- Agoraphobia (fear and avoiding places or situations that might cause panic and feelings of being trapped, helpless, or embarrassed)
- Anger management or difficulty controlling your anger
- Anxiety disorder
- Attention deficit disorder (ADD)
- Attention deficit hyperactivity disorder (ADHD)
- Bipolar depression
- Brain fog (disturbance of memory, word-finding difficulties, difficulties in concentrating)
- Depression
- Dystonia (movement of the body that you cannot control)
- Fainting or near fainting
- Fatigue (significant, on a regular basis)
- Hallucinations
- Migraine-like headaches
- Light and/or sun sensitivity
- Mania or hypomania
- Muscle pain or tenderness
- Muscle tightness caused by hyperventilation
- Muscle weakness in the legs or arms
- Nerve pain, numbness, tingling in the legs or arms
- Obsessive compulsive disorder (OCD)
- Increased sensitivity to feeling pain and/or an extreme response to pain
- Panic attacks (these may have occurred as a teenager or child)
- Post-traumatic stress syndrome or disorder (PTSD)
- Psychosis or schizophrenia
- Restless legs syndrome (the compelling urge to move your legs or arms while at rest, often associated with discomfort, usually at night and at rest, temporarily improves with movement. It is not muscular cramps or resulting from pain in the back)
- Seizure-like activity
- Insomnia
- Irresistible drowsiness/sleep attacks
- Significant sensitivity to odors/scents (e.g., nausea, headache, or passing out)
- Loss of the ability to smell
- Ringing or other noises in the ear(s) bother you and were not caused by loud noise injury and do not sound like pulsations
- Sensitivity to noises that most people do not hear and bothers you a lot
- Acoustic startle (muscular activity in response to a sudden loud sound)
- Tourette's syndrome with verbal or movement tics (now or in the past)
- Tremor at rest
- Other problems - please list here: ___
- None of the above: ___
- I am not sure since I was on other medicines at the time: ___

5. Did you have side effects from antihistamines? Yes/No

6. If yes, did the side effects resolve over time while continuing the antihistamines? Yes/No

7. Please write in side effects:___

8. Did the side effects make you stop taking them? Yes/No
