# Supplementary material for: Prevalence and treatment response of neuropsychiatric disorders in mast cell activation syndrome
Source: Brain Behav Immun Health. 2025 Jun 30;48:101048. doi: 10.1016/j.bbih.2025.101048 (PMC12270938; doi:10.1016/j.bbih.2025.101048)
Supplement: Multimedia component 2 [file mmc2.docx]

**Mast cell mediator release syndrome questionnaire [2025 version]**

Name: ______________________________ Birth date: _________ Date: _________

**Check the following symptoms that you experienced in the past and now:**

**CHILDHOOD**

|  | Abdominal pain |  |  | Constipation |  |  | Hives |  |  | Nausea |
| --- | --- | --- | --- | --- | --- | --- | --- | --- | --- | --- |
|  | Anaphylaxis |  |  | Eczema |  |  | Infections: odd or frequent |  |  | Rashes |
|  | Asthma |  |  | Food allergies |  |  | Insect reactions |  |  | Seasonal allergies:  severe |
|  | Colic |  |  | Headaches |  |  | Migraines |  |  | Vaccine reactions: severe |

(Anaphylaxis attack is defined as having swelling, tight throat, and wheezing)

**TEENAGE**

|  | Abdominal pain |  |  | Constipation |  |  | Headaches |  |  | Migraines |
| --- | --- | --- | --- | --- | --- | --- | --- | --- | --- | --- |
|  | Anaphylaxis |  |  | Diarrhea |  |  | Head trauma |  |  | Prolonged infectious mono |
|  | Asthma |  |  | Fainting or near faint |  |  | Hives |  |  | Seasonal allergies: severe |
|  | Bruising |  |  | Food reactions |  |  | Insect reactions |  |  | Vaccine reactions: severe |

Females: Menstruation: severe, painful, or heavy Endometriosis

**ADULT**

|  | Abdominal pain |  |  | Constipation |  |  | Headaches  or migraines |  |  | Poor healing |
| --- | --- | --- | --- | --- | --- | --- | --- | --- | --- | --- |
|  | Anaphylaxis |  |  | Diarrhea |  |  | Hives |  |  | Ringing/sounds in ears |
|  | Asthma |  |  | Fainting or near faint |  |  | Insect reactions |  |  | Seasonal allergies: severe |
|  | Bruising |  |  | Food reactions |  |  | Medicine allergy/reactions |  |  | Vaccine reactions: severe |

Panic attacks Anxiety Depression ADHD Tremors Edema

Odor or scent reactions: nausea, headache, lightheaded

Restless legs: nightly compelling urge to move legs while awake, usually associated with

discomfort, and moving or walking helps

Weight issues: too thin or too heavy with inability to lose weight

Females: Endometriosis Polycystic ovary syndrome Miscarriages

**Serious triggers** (See Trigger Table for all triggers)

Chemical exposure Metal dental fillings

Mold exposure Tick bites Metal, plastic, or foreign body implants

**FAMILY HISTORY:** Relatives with unusual syndromes/undiagnosed illnesses: **yes** or **no**

**________________________________________________________________________________**

Answer the following even if they occurred in the past, slightly bothersome, rarely occurred, or may seem not related to your main problems. If the statement applies to you, check the box, and then enter the severity level on the line next to the box. The number should be graded when it was present the last time it occurred. If the statement does not apply, do not check the box, or enter a number.

Use the range of 1 (very mild) to 10 (unbearable) to reflect the severity level of your discomfort.

✓

**Example: __6__** The following symptoms occur repeatedly or may be constant. Do not score other areas marked for the staff to score.

**GENERAL HEALTH** [For staff: score 1 for each if present]

Do you get colds regularly which then turn into bacterial infections such as bronchitis or sinus infections? ……………………………………………………………………...Yes___ No___

Has the course of your illness been episodic (and/or with attacks)? ........Yes___ No___

Have the symptom-free periods become shorter and shorter? …………..Yes___ No___

**Applies Severity**

Significant physical weakness or fatigue doing everyday activities….

Extreme fatigue attacks, it can be hard to keep eyes open ……….…

At times I lose weight despite maintaining my normal diet …………..

**EYES, EARS, NOSE, MOUTH** [For staff: score 1 if one or more are present]

Ears have ringing or odd sounds ………………………….…………..

Eyes are dry, itchy, red, burning, or feel gritty ……………………….

Runny or stuffy nose ……………………………………………………

Inflammation or ulcers of the mouth …………………………………

**CHEST and HEART**

[For staff: score 1 for each if present]

Burning and/or pressure pain in the chest…………………………..… (Normal electrocardiogram or stress test; or not severe enough to go to ER)

Rapid heart rate (rapid palpitations) …………………………….………

…………………………………………………………………………………………………

[For staff: score 2 for each of the following three symptoms if present]

Redness or flushing of the skin, especially face or upper body ……..

Hot flashes (usually with dry skin lasting 2 to 5 minutes, rarely 10 minutes and often occur with nausea or other symptoms. …….....

(these are not menopausal hot flashes with wet sweats)

Sudden dizziness/lightheadedness with fainting or near faint ….........

**LUNGS** [For staff: score 1 if one or more is present]

Irritable dry cough or need to cough …………………………………….

Feeling of shortness of breath or difficulty taking a full breath ……….

Asthma-like complaints (wheezing) ……………………………………..

**ABDOMEN** [For staff: score 1 for each of the following if present]

Attacks of visible bloating or distension within minutes....…….………

Pain in the abdomen …………………………….……………………..…

Pain is burning …………………………………………………………….

Pain is crampy or spastic ………………………………………………...

Pain is associated with diarrhea (watery or loose stool) ……………...

Nausea (with or without vomiting) ……………………….……..……….

Antihistamines reduce nausea (Allegra, Benadryl, cetirizine, Claritin, diphenhydramine, fexofenadine, loratadine, Xyzal, Zyrtec)? ………… Yes ___ No ____

(This does not include specific nausea medicine like prochlorperazine, ondansetron, or Zofran)

Have you had abdominal adhesions without prior surgery or infection? …………..Yes ___ No ____

**URINE/PELVIS** [For staff: score 1 if present]

Bladder and/or pelvic pain (this applies to both women and men). Often associated with painful, frequent, and/or urgent urination,

and may be associated with pain during sexual intercourse..…….…….

(during these times bacterial cultures and urine analysis are normal)

**NEUROLOGIC and MUSCULOSKELETAL** [For staff: score 1 for each of the following if present]

Migraine-like headaches (throbbing on one side only and/or diagnosed

as a migraine. These are not tension headaches)……………………….

Brain fog – word finding problems and/or concentration difficulties with

or without associated insomnia episodes ………..……………………….

Leg or arm pain and/or altered feelings including, numbness, tingling,

burning, sharp pain, and pins and needles. ………………………………

(this pain does not respond to over-the-counter medicine)

Muscle pain or tenderness [Does not count in total point system] …….[ ___ ]

**SKIN** [For staff: score 1 for each of the following if present]

See the photographic examples below.

Hives (red raised itchy spots) ……………………………………………….

Hemangiomas (raised or flat bright red spots)…………………….............

During attacks there are itchy skin lesions that look like acne in the corners of the nasal-lip area, chin, and forehead ….……………………...

Knots, nodules, cysts under the skin (including cystic acne)..…………..

Painless, non-itchy swelling (especially lips, cheeks, eyelids) …………...

Itching in area around the anus during attacks……………………………..

Itching without a rash [this does not count in total point system] ……...…[ __ ] _______

**HEMATOLOGIC** [For staff: score 1 if one or more is present]

Bruising after minor injuries …………………………….…………………….

Unusual nose bleeds ………………………………………………………….

Women only: significant menstrual bleeding ………..……………………….

**TRIGGERS** [For staff: score 1 for each if positive]

**Are any of the symptoms or complaints listed above worsened by:**

High histamine foods (see examples below)…………………………………

Sleep deprivation (being awake for more than 24 hours)...................................

Hunger or fasting (having no food all day)...................................................

**BONE** [For staff: score 1 if one or more is present]

Bone pain that usually occurs in more than one bone …………………..……

Osteoporosis or osteopenia or osteosclerosis of unknown cause ……………

Examples of skin changes:

Hemangiomas Acne during attacks Bruises


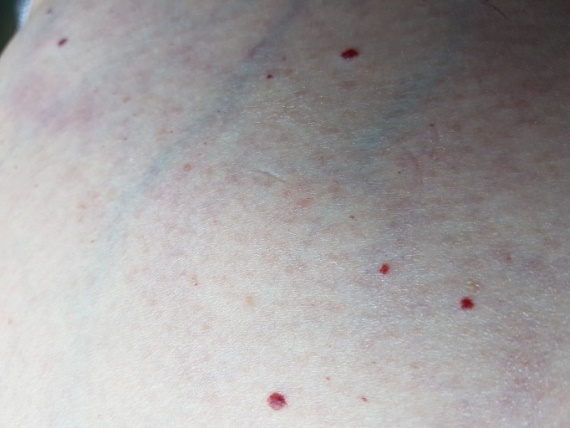

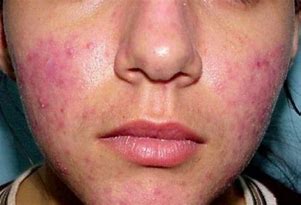
  
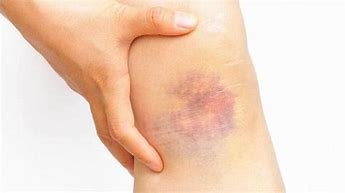


Flushing Hives Nodules Swelling


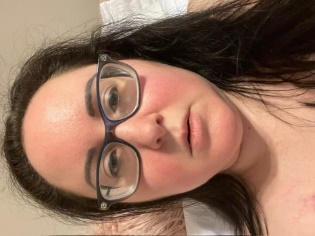

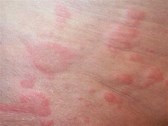

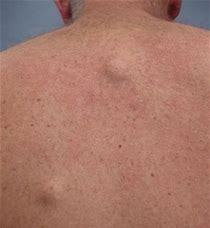

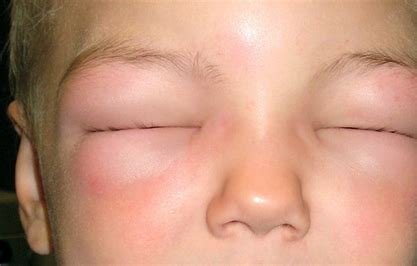


**Examples of high histamine foods:** wine, beer, chocolate, coffee, tea, tomatoes, cherries, cured meat (salami), cured fish, left-over meat, processed meat, peanuts, beans, shellfish, aged cheese, avocado, vinegar, bone broth, fermented dairy, vegetables, and grain.

**For staff only:**

**Laboratory Data**

At least once during the disease phases there was:

**Applies**

Hyperbilirubinemia up to about 2.5 mg% with the exclusion of

Meulengracht/Gilbert’s syndrome or another hereditary disorders □

Increase in transaminases:

γGT and/or □

ALT and/or □

AST and/or  □

Score 1 if one or more is present. □ **1**

*AST increased >10 fold (subtract 1 point and look for liver diseases) □ -****1***

Hypercholesterolemia (patient must be normal or underweight) □ **1**

Low titer autoantibodies without a corresponding organ symptom □ **1**

**Mast cell mediators:**

Tryptase in serum was normal □ **0**

Tryptase was increased <2 times the upper limit □ **3**

Tryptase increased >2 times the upper limit □ **10**

Histamine in plasma was normal □ **0**

Histamine was increased <2 times the upper limit □ **3**

Histamine increased >2 times the upper limit □ **10**

Prostaglandin D2 in plasma was normal □ **0**

Prostaglandin D2 was increased <2 times the upper limit □ **3**

Prostaglandin D2 increased >2 times the upper limit □ **10**

Heparin and/or factor VIII in plasma was/were normal □ **0**

Heparin and/or factor VIII was/were elevated (and bleeding disorders were excluded). □ **3**

Chromogranin-A in serum was normal □ **0**

Chromogranin-A was increased (and other causes were excluded) □ **3**

Leukotriene E-4 in urine was normal □ **0**

Leukotriene E-4 was increased < 10 times the upper limit □ **1**

Leukotriene E-4 was 10 times the upper limit □ **5**

Leukotriene E-4 was >10 times the normal limit □ **10**

N-methylhistamine in urine was normal □ **0**

N-methylhistamine was increased < 10 times the upper limit □ **1**

N-methylhistamine was 10 times the upper limit □ **5**

N-methylhistamine was >10 times the normal limit □ **10**

2,3 dinor 11b PG F2 alpha in urine was normal □ **0**

2,3 dinor 11b PG F2 alpha was increased 10 times the upper limit□ **1**

2,3 dinor 11b PG F2 alpha was 10 times the upper limit □ **5**

2,3 dinor 11b PG F2 alpha was >10 times the normal limit □ **10**

Other conspicuous laboratory findings (please name with values) □ **0**

_________________________________________________________________________________

**Procedures and Imaging**

Esophagogastroduodenoscopy or associated biopsies had:

no pathological findings  □ **0**

or

mild inflammation □ **1**

or

Helicobacter pylori-negative and NSAID-negative erosions

and/or ulcers □ **3**

or

diffuse and/or focal mast cell infiltrates ≥20/hpf with rounded shape □ **5**

or

Mast cell nests and/or sheets of spindle-shaped mast cells

and/or CD25-positive mast cells □ **10**

Colonoscopy and associated biopsies had:

no pathological findings □ **0**

or

mild inflammation □ **1**

or

focal and/or disseminated dense infiltrates of

morphologically inconspicuous mast cells □ **5** or Mast cell nests and/or sheets of spindle-shaped mast cells and/or CD25-positive mast cells □ **10**

Enlargement of the spleen □

and/or enlargement of the liver □

□ **1**

Diseases and disorders below should be excluded in order help confirm the presence of a mast cell disorder. Symptoms in organ/tissue systems can be similar in both. Evaluate both checklists and the numerical values ​​listed to the right of each box. Add together to get a sum.

**Sum 9 to 13** = pathological activation of mast cells as cause of complaint is assumed.

**Sum ≥14** = diagnosis of mast cell mediator release syndrome is clinically confirmed.

**Sum of points: ______** **Diagnosis: mast cell mediator release syndrome**

**5 or more systems involved may also be important.**

**Differential diagnosis and testing for disorders that are multi-systemic diseases, present with similar symptoms, or exacerbate mast cell activation**

**Endocrine disorders** – all are multi-systemic

Adrenal insufficiency (cosyntropin stimulation test)

Diabetes mellitus (history and labs – Type 1 or 2 with systemic complications)

Endometriosis (gynecologic exam, laparoscopy)

Fabry disease (neuropathic pain, fatigue, angiokeratomas, cloudy cornea, genetic test)

Porphyria (lab: spot PBG urine)

Thyroid disorders – multi-systemic and can exacerbate MCAS (labs: thyroid panel)

**Gastrointestinal disorders**

Amyloidosis – multi-systemic (fat biopsy, rectal biopsy)

Celiac disease – multi-systemic and can exacerbate MCAS (antibody studies, endoscopic biopsy)

Cholecystitis (ultrasound, DISIDA imaging)

Chronic bacterial infection or post-infectious autoimmune state (fecal PCR, lactulose breath test) – multi-systemic and may exacerbate MCAS (endoscopic biopsy, urea breath test, antibody study)

Hepatitis multi-systemic (hepatitis profile, other laboratory tests)

Inflammatory bowel disease – multi-systemic (colonoscopy/ileoscopy and biopsy; video capsule endoscopy)

Lactose, sucrose, or fructose intolerance as cause of bloating and altered bowel habits (history, specific breath tests)

Median arcuate ligament syndrome – multi-system and can exacerbate MCAS (auscultation, CT angiography with deep expiration views)

Microscopic colitis associated with celiac disease or cryptosporidiosis - multi-systemic (colonoscopy biopsy, cryptosporidiosis antigen)

Parasitic infection – multi-systemic (fecal antigen testing, ova and parasite exam, PCR testing)

Small bowel obstruction with and without small intestinal bacterial overgrowth – multi-systemic (adhesions, volvulus, hernia, tumors) (history, physical, imaging studies, lactulose breath testing)

Small intestinal bacterial overgrowth – multi-systemic and exacerbates MCAS (lactulose breath test, glucose breath test, duodenal or jejunal aspirate culture)

**Immunological, Inflammatory and Rheumatologic diseases** – all multi-systemic

Asthma and atopic diseases including chronic urticaria (history, allergy testing – skin prick and RAST – MC mediators, ESR, CRP, TFT, ANA, H. pylori testing, AST, stool tests for parasites and fungus)

Alpha-gal syndrome – mammalian meat allergy, hives, allergies, GI symptoms

Chronic fatigue syndrome (history)

Chronic pelvic pain syndromes [interstitial cystitis, type III chronic prostatitis, vulvodynia] (cystoscopy and biopsy, prostatic secretion testing)

Familial Mediterranean fever (history, family history, ESR)

Fever of unknown etiology. (history, extensive lab testing)

Fibromyalgia (history, physical, ESR)

Food allergy/sensitivity (history, skin prick tests, RAST, special investigations of biopsies, elimination diet)

Heredity alpha 1 tryptasemia (tryptase level, genetic testing)

Hypereosinophilic syndrome (CBC with differential)

Hypermobile Ehlers Danlos syndrome (history, physical exam)

Hereditary angioedema (family history, C4 level, C1-esterase inhibitor level)

Juvenile rheumatoid arthritis (history, RF, ESR, radiographs)

Lupus erythematosus (history, ANA, ESR)

Sjogren’s disease (antibody studies including novel or early Sjogren’s antibodies)

Vasculitis (clinical picture, ANA, ESR, and other laboratory studies)

**Infectious diseases** – all multi-systemic

*Bartonella - can also exacerbate MCAS (*PCR and FISH tests*)*

Mold infection - chronic inflammatory response syndrome - can also exacerbate MCAS (urine aflatoxin test and serum antibody tests)

Syphilis - can also exacerbate MCAS

Tick borne infections - can also exacerbate MCAS (antibody panel)

Tuberculosis (blood and skin tests, radiographs)

**Neoplastic diseases**

Carcinoid tumor – multi-systemic (24-hour urine 5-HIAA, CT and octreotide imaging)

Intestinal lymphomas multi-systemic (CT studies, endoscopic procedures)

Pancreatic endocrine tumors [gastrinoma, insulinoma, glucagonoma, somatostatin, VIPoma] – multi-systemic (lab determination, imaging studies, endoscopic ultrasound)

Pheochromocytoma – multi-systemic (laboratory studies, CT imaging)

**Neurologic and Psychologic diseases**

Cyclic vomiting syndrome (history)

Migraine (history)

Munchausen’s syndrome (history)

Postural orthostatic tachycardia syndrome – multi-systemic and exacerbates MCAS (orthostatic pulse exam, tilt table test, autonomic testing)

Serotonin syndrome (history, serotonin level)

Small fiber peripheral neuropathy – multi-systemic (skin biopsy)
